# Effects of the combination of P3-based GKT and reality monitoring on deceptive classification

**DOI:** 10.3389/fnhum.2013.00018

**Published:** 2013-01-31

**Authors:** Ki-Won Jang, Deok-Yong Kim, Sungkun Cho, Jang-Han Lee

**Affiliations:** Clinical Neuro-psychology Laboratory, Department of Psychology, Chung-Ang UniversitySeoul, South Korea

**Keywords:** lie detection, Guilty Knowledge Test, reality monitoring, P3, leakage of knowledge

## Abstract

The study aimed to investigate whether a combination of the P3-based Guilty Knowledge Test (GKT) and reality monitoring (RM) distinguished between individuals who are guilty, witnesses, or informed, and using both tests provided more accurate information than did the use of either measure alone. Participants consisted of 45 males that were randomly and evenly assigned to three groups (i.e., guilty, witness, and informed). The guilty group conducted a mock crime where they intentionally crashed their vehicle into another vehicle in a virtual environment (VE). As those in the witness group drove their own vehicles, they observed the guilty groups' vehicle crash into another vehicle. The informed group read an account and saw screenshots of the accident. All participants were instructed to insist that they were innocent. Subsequently, they performed the P3-based GKT and wrote an account of the accident for the RM analysis. A higher P3 amplitude corresponded to how well the participants recognized the presented stimulus, and a higher RM score corresponded to how well the participants reported vivid sensory information and how much less they reported uncertain information. Findings for the P3-based GKT indicated that the informed group showed lower P3 amplitude when presented with the probe stimulus than did the guilty and witness groups. Regarding the RM analysis, the informed group obtained higher RM scores on visual, temporal, and spatial details and lower scores on cognitive operations than the guilty and witness groups. Finally, discriminant analysis revealed that the combination of the P3-based GKT and RM more accurately distinguished between the three groups than the use of either measure alone. The findings suggest that RM may build upon a weakness of the P3-based GKT's. More specifically, it may build upon its susceptibility to the leakage of information about the crime, therefore helping protect innocent individuals who have information about a crime from being perceived as guilty.

## Introduction

Deception occurs in a variety of interpersonal situations. Individuals are able to detect deception using several methods. When an individual tells a lie, that person unconsciously displays potential cues that may be behavioral, verbal, and psychophysiological (Vrij, 2000; Gamer et al., 2006). Typically, lie detection tools are designed to detect a lie using these cues. One commonly used lie detection tool is the arousal-based polygraph (Vrij, 2000). The arousal-based polygraph detects a lie based on differences in psychophysiological responses (e.g., electrodermal response, cardiovascular, and respiratory) to crime-relevant and crime-irrelevant questions (Kircher and Raskin, 1988; Richardson et al., 1990; Ben-Shakhar et al., 1999). Despite its usefulness in the field, the arousal-based polygraph has some limitations. For example, the arousal-based polygraph indirectly detects a lie by measuring variables related to the lie (e.g., guilty, anxiety). However, these emotions can appear not only in a situation where individuals tell a lie but also in an uncertain situation where innocent individuals are false accused (Allen and Mertens, 2009).

Another method of lie detection is based on recognition of crime-related information that is stored in memory. This method is called the Guilty Knowledge Test (GKT). Individuals who commit a crime have specific knowledge or memories about the crime, whereas innocent individuals do not. The GKT examines whether suspects possess this specific knowledge. If a guilty suspect recognizes the crime-related evidence presented, he or she is more likely to produce higher physiological responses than will non-guilty suspects (Vrij, 2000). One of the methods used to detect such recognition is the event-related potentials (ERPs). The ERPs provides considerably accurate information regarding temporal changes in brain activity in response to the processing of a particular stimulus. Of the ERPs components, the P300 (P3) component is evoked in response to attentive, recognized, and meaningful stimuli (Polich and Kok, 1995; Polich, 2000).

The P3-based GKT is a tool that uses temporal changes in brain activity to detect deception (Farwell and Donchin, 1991; Rosenfeld et al., 1991; Allen et al., 1992; Abootalebi et al., 2006). In the P3-based GKT, three types of stimuli, target, probe, and irrelevant, are presented to participants. Probe stimuli contain concealed crime-related information that only the guilty individuals possess (Ben-Shakhar and Elaad, 2002). Irrelevant stimuli are stimuli with information that is unrelated to the crime. Target stimuli are also stimuli that contain information unrelated to the crime. Target stimuli are essentially a list of stimuli the participants are instructed to respond to by pressing a button when the stimuli are presented (Farwell and Donchin, 1991). The basic assumption of the P3-based GKT is that guilty individuals will recognize the probe stimuli, thus evoking higher P3 amplitude in their brain potentials compared to that evoked by the irrelevant stimuli. Conversely, innocent individuals will show no differences in P3 amplitude in brain potentials in response to the probe and irrelevant stimuli. Prior studies of ERPs-based GKT demonstrated that accurate rates of deception detection are relatively high, ranging from 89 to 90% (Farwell and Donchin, 1991; Rosenfeld et al., 1991; Allen et al., 1992). Despite its usefulness, the P3-based GKT is weak in natural environments. For example, probe stimuli may be disclosed to the public through mass media or disseminated verbally by individuals participating in the investigation (e.g., a criminal investigator). The witnesses may also have information about the crime even though they did not conduct a crime. Thus, they may not be classified into the innocent groups because of their knowledge or memories of the crime, which discourages the use of the P3-based GKT (Ben-Shakhar et al., 1999).

The quality of information about the crime may be different for guilty vs. innocent individuals even though they both have information about a particular crime. Given that they conducted the crime, guilty individuals may have more vivid sensory information about the crime as compared to innocent individuals. Such differences can be revealed by reality monitoring (RM), one type of statement analyses conducted in a crime setting. RM is based on differences in the quality of information contained in an individual's memory for experienced vs. imagined events (Johnson and Raye, 1981; Memon et al., 2010). The assumption of RM is that experienced memories differ from fictional memories (e.g., Vrij et al., 2001). The RM evaluates the quality of information contained in memories using several criteria, including visual, auditory, temporal, and spatial details as well as cognitive operations. The visual and auditory details involve perceptual and sensory information. The temporal details provide information about timing or duration of events. The spatial details include information regarding where the event took place and how objects and people were situated in relation to each other. Memories of experienced events may involve more perceptual, sensory, spatial, and temporal details than the memories of imagined events. However, imagined memories that are obtained through an internal source are likely to contain thoughts and reasoning in one's testimony, called cognitive operations. Cognitive operations are usually vague and not concrete (Vrij, 2008) and therefore are less frequent in memories of the experienced events as compared to memories of imagined events (Vrij et al., 2004). In criminal situations, an individual's statement about an experienced event is likely to contain the truth, whereas a statement pertaining to an imagined event is likely to contain false information (Vrij, 2008). Thus, the RM analysis would differentiate statements between those who actually experience a crime (guilty individuals) and those who receive information about the crime only (witness or informed individuals). By distinguishing between those who have experienced a crime and those who only have information about a crime, RM can determine whether individuals are innocent. The individuals who witnessed the crime are more likely to describe perceptual and sensory details of the experienced event in their statements, whereas the guilty and informed individuals may describe the imagined event without actually experiencing it. For example, the guilty and informed individuals are more likely to make up a story either because they did not actually experience the crime or because had to pretend being innocent. Given these, RM may be used to complement and overcome a possible weakness of the P3-based GKT.

The aim of this study was to investigate whether a combination of the P3-based GKT and RM would more effectively differentiate among guilty individuals who conduct a crime, witnesses who experience the crime, and informed individuals who did not experience the crime but have information about the crime. Several hypotheses guide the current study. First, we predicted that in the P3-based GKT, the guilty group would show higher P3 amplitude in response to the probe stimuli than the other two groups. Second, we predicted that the witness group would meet the RM criteria more frequently than the other two groups. Third, we predicted that the combination of the P3-based GKT and RM would discriminate the groups more accurately than the P3-based GKT or RM alone.

## Materials and methods

The participants consisted of 45 male undergraduates (15 per group), and the mean age of the sample was 23.07 years (SD = 2.41). They were randomly and evenly assigned to three groups: guilty, witness, and informed. This study used male participants because gender may affect ERPs amplitude (e.g., Cahill and Polich, 1992; Polich and Martin, 1992; Reinvang, 1999). We used the Machiavellianism Scale (Christie and Geis, 1970), the Social Adroitness Scale (Jackson, 1994), and Levenson's Self-Report Psychopathy Scale (LSRP) (Levenson et al., 1995) to control for the manipulativeness of the participants. No significant differences were found among groups; the Machiavellianism Scale *F*_(2, 42)_ = 0.68, *n.s*.; the Social Adroitness Scale *F*_(2, 42)_ = 0.43, *n.s*.; the LSRP *F*_(2, 42)_ = 0.44, *n.s*. (Table 1).

**Table 1:** **Demographic data and the questionnaire scores**.

	**Mean (SD)**		
	**Guilty group**	**Witness group**	**Informed group**
	**(*n* = 15)**	**(*n* = 15)**	**(*n* = 15)**
Age	23.27 (1.94)	22.93 (2.84)	23.00 (2.51)
Machiavellianism	50.87 (5.18)	51.13 (6.10)	48.80 (6.68)
Social Adroitness	12.60 (2.32)	11.67 (3.39)	11.87 (2.85)
LSRP	36.47 (6.97)	37.60 (6.58)	35.40 (5.65)

LSRP, Levenson's self-report psychopathy scale.

A 3D visual display was presented on dual monitors through an Olympus FMD-250W head-mounted display with a resolution of 800 × 600 pixels. A computer game (Grand Theft Auto San Andreas: GTASA) that involved a third-person shooter and a driving simulator in a virtual environment (VE) was used.

When the participants arrived at the laboratory, they were asked to sign a consent form and then the experimenter explained the objectives of the study. Each participant was randomly assigned to one of the groups (i.e., guilty, witness, or informed). The guilty or witness group wore a head-mounted device while experiencing the VE. Two vehicles were driven in the VE. One vehicle was driven by the guilty or witness group, and the other one was driven by an experimenter. The guilty group intentionally crashed their vehicle into the experimenter's vehicle (i.e., head-on collision). The crash was severe enough to blow the hood off the vehicles. The witness group was instructed to drive safely and watch the crash that was caused by the experimenter. The informed group was instructed to watch screenshots of the car accident caused by the guilty group and read the description of the accident. The duration of the VE was approximately 15 min. All of the groups were instructed to write statements about the car accident and to insist that they were innocent.

Subsequently, all participants were given the GKT to evaluate whether they recognized the vehicle of assailant. Three types of stimuli (irrelevant, probe, and target stimuli) were used in the GKT. The irrelevant stimuli consisted of four screenshots of a vehicle not related to the accident, and the probe stimulus was a screenshot of the assailant's vehicle. All participants were instructed to respond by pressing a spacebar when the target stimulus (i.e., a vehicle unrelated to the car accident) was presented. The vehicles used in this experiment were similar in size and color but different in shape. Each trial consisted of one probe, four irrelevant, and one target stimuli (total 40 trials). After presenting a fixation for 500 ms, the probe, irrelevant, and target stimuli were randomly presented for 1000 ms, and then a blank screen followed for 1500 ms with an inter-stimulus-interval (ISI) of 3000 ms (visual angle being 16° in width and 13° in height; 518 × 370 pixels). After attaching the electrodes to the head of each participant, the participants were instructed to press a “yes” button when the target stimuli were presented and a “no” button when the others were presented. The total duration of the P3-based GKT was approximately 12 min.

EEG data were recorded from 28 sites (Fpz, Fz, FCz, Cz, CPz, Pz, Oz, Fp2, F3/4, F7/8, FC3/4, C3/4, CP3/4, P3/4, P7/8, O1/2, T7/8, and FT7/8) with reference electrodes on the earlobes using the Neuroscan System (Neuroscan Labs, Sterling, VA, USA). EOGs were recorded from the outer canthus of each eye to measure horizontal electro-oculograms (HEOG) activity and from the left eye to measure vertical electro-oculograms (VEOG) activity. All impedances were maintained at 5 kΩ or less. The data were digitized at a rate of 512 Hz for 800 ms and recorded with a bandpass of 0.01–100 Hz. Epochs were created from -100 to 898 ms around the stimulus and baseline corrected using the 100 ms pre-stimulus period. Artifacts in which the EEG or EOG exceeded ±100 μV were rejected (Semlitsch et al., 1986). The bandpass filter was applied 0.05–10 Hz (24 dB octave/slope). The P3 component was typically defined as the largest positive peak occurring between 300 and 1000 ms at each electrode (Abootalebi et al., 2006). Amplitude was measured as the difference between the mean pre-stimulus baseline and maximum peak amplitude at Pz, because the Pz site is usually reported to be maximal among the other sites. Peak detection was done automatically but verified manually.

The RM analysis consisted of five domains: visual, auditory, temporal, and spatial details as well as cognitive operations. First, a statement met the criteria of having visual details if it contained a vivid or clear description. Second, a statement met the criteria for having auditory details if it encompassed auditory information. Third, a statement met the criteria for temporal details if it included the order in which the accident occurred. Fourth, a statement met the criteria for spatial details if the statement encompassed locational information on humans or objects. Fifth, a statement met the criteria for cognitive operations if it contained descriptions of imagined events from internal source, such as thoughts and reasoning. Cognitive operations were scored dichotomously: a score of 0 when cognitive operations were not present and a score of 1 when cognitive operations were presented. For the RM, a score for each domain was calculated by summing of frequency of meeting the criteria in their statements by two independent raters (Vrij et al., 2004).

## Results

### P3-based GKT

**Figure 1:**
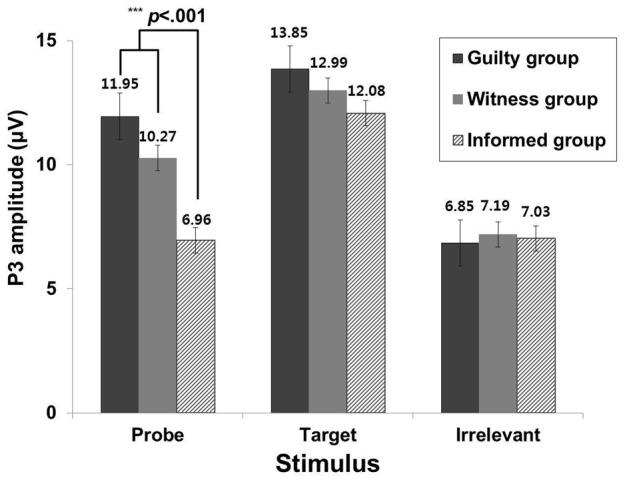
**The difference of P3 amplitude among the groups on each stimulus condition**.

**Figure 2:**
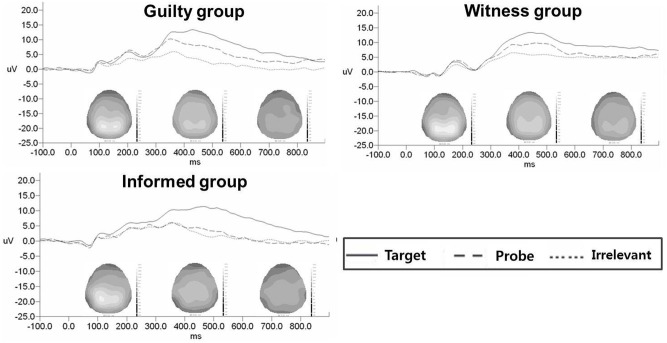
**The grand averages among the groups for superimposed probe, target, and irrelevant stimulus**.

A 3 (group: guilty, witness, and informed) × 3 (stimulus: probe, irrelevant, and target) repeated-measures ANOVA was performed to analyze the P3 data. The results indicated that there was a significant interaction effect between group and stimulus, *F*_(4, 84)_ = 3.27, *p* < 0.05, η^2^ = 0.14. Subsequently, we performed One-Way ANOVA for each stimulus to investigate differences among the groups. There was a significant main effect of group membership on the P3 amplitudes for the probe stimuli, *F*_(2, 42)_ = 8.42, *p* = 0.001, η^2^ = 0.29, but there were no significant group differences for the target and irrelevant stimuli, *F*_(2, 42)_ = 0.31; *F*_(2, 42)_ = 0.37, *n.s*. In the pairwise comparison test, the informed group showed lower P3 amplitudes in response to the probe stimuli than the guilty, *t*_(28)_ = 4.13, *p* < 0.001, and witness groups, *t*_(28)_ = 2.68, *p* = 0.01. The difference between the guilty and the witness groups, however, did not reach statistical significance, *t*_(28)_ = −1.33, *n.s*. (Figure 1). Additionally, there was a significant main effect of stimulus, *F*_(2, 84)_ = 49.48, *p* < 0.001, η^2^ = 0.54. A pairwise comparison test indicated that P3 amplitudes in response to the target stimuli were higher than those in response to the probe, *t*_(44)_ = 4.43, *p* < 0.001, and irrelevant stimuli, *t*_(44)_ = 8.99, *p* < 0.001, and that the P3 amplitudes in response to the probe stimuli were significantly higher than the irrelevant stimuli, *t*_(44)_ = 5.89, *p* < 0.001. However, there was no main effect of group (Figure 2).

### Reality monitoring

**Figure 3:**
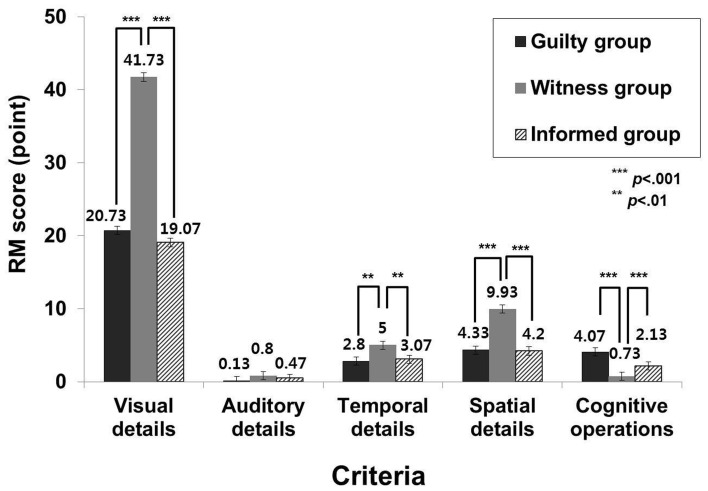
**The difference of the RM scores among the groups on each criterion**.

For the RM analysis, a 2 (group: guilty, witness, and informed) × 5 (criteria: visual, auditory, temporal, spatial, and cognitive operations) MANOVA was used. A MANOVA revealed a significant multivariate main effect for group, Wilks' = 0.13, *F*_(10, 76)_ = 13.61, η^2^ = 0.64. At a univariate level, there were significant main effects for visual, temporal, and spatial details as well as cognitive operations: visual details, *F*_(2, 42)_ = 57.10, *p* < 0.001, η^2 = 0.73^; temporal details, *F*_(2, 42)_ = 6.43, *p* < 0.01, η^2^ = 0.23; spatial details, *F*_(2, 42)_ = 12.57, *p* < 0.001, η^2^ = 0.37; and cognitive operations, *F*_(2, 42)_ = 34.20, *p* < 0.001, η^2^ = 0.62. A pairwise comparison tests indicated that the witness group reported significantly more visual, temporal, and spatial details than did the guilty [visual details: *t*_(28)_ = 8.70, *p* < 0.001; temporal details: *t*_(28)_ = 2.84, *p* < 0.01; spatial details: *t*_(28)_ = 3.72, *p* = 0.001] and informed groups [visual details: *t*_(28)_ = 8.87, *p* < 0.001; temporal details: *t*_(28)_ = 3.04, *p* < 0.01; spatial details: *t*_(28)_ = 3.80, *p* = 0.001]. In terms of the auditory details, however, there were no significant differences among the groups. For cognitive operations, the witness group had significantly less than the guilty, *t*_(28)_ = −8.30, *p* < 0.001, and informed group, *t*_(28)_ = −3.55, *p* = 0.001. Furthermore, the informed group also reported cognitive operations significantly less than the guilty group, *t*_(28)_ = −4.63, *p* < 0.001 (Figure 3).

### Discriminant analyses for P3-based GKT and reality monitoring

A discriminant analysis was conducted to investigate whether a combination of the P3-based GKT and RM was better at distinguishing between the guilty, witness, and informed groups than the P3-based GKT (i.e., probe, irrelevant, and target stimuli) or RM (i.e., visual, auditory, temporal, and spatial details as well as cognitive operations). To develop an optimum classifier to discriminate among the groups, two discriminant analyses were conducted. One analysis was performed to find a discriminant function to maximize the separation between the guilty and witness groups, and the other analysis was performed to distinguish between the guilty and informed groups.

First, we conducted a discriminant analysis to determine whether the P3-based GKT, RM, and a combination of the P3-based GKT and RM distinguished between the guilty and witness groups. For the results of the P3-based GKT, univariate *F* tests showed that the discriminant function was not significant, χ^2^_(3, *N* = 30)_ = 5.20, *p* = 0.16, and indicated that 82.2% of total variance was not explained, λ = 0.82. In the RM, univariate *F* tests indicated significant differences for the visual, auditory, temporal, and spatial details and cognitive operations, χ^2^_(5, *N* = 30)_ = 49.93, *p* < 0.001. The mean classification accuracy was 100.0%. Table 2 shows that 100.0% of the guilty group and 100.0% of witness group were classified correctly in the present study. An internal validation of the discriminant analysis (jackknife) also indicated 100.0% correct classifications. The result for the combination of P3-based GKT and RM showed significant differences for the predicted variables, χ^2^_(8, *N* = 30)_ = 48.60, *p* < 0.001. The mean classification accuracy was 100.0%. In these analyses, 100.0% of the guilty group and 100.0% of the witness group were classified correctly both in the present study and in the internal validation of the discriminant analysis (Table 2).

**Table 2:** **Discriminant analyses with P3-based GKT and reality monitoring between the guilty and witness group**.

**Cross validated classification rates (original classification)**					
	**Hit rates**			**Eigenvalue**	**λ**
	**Guilty group**	**Witness group**	**Total**		
P3-GKT	60.0 (73.3)	40.0 (26.7)	73.3 (80.0)	0.22	0.82
RM	100.0 (100.0)	100.0 (100.0)	100.0 (100.0)	6.09	0.14
P3-GKT + RM	100.0 (100.0)	100.0 (100.0)	100.0 (100.0)	6.58	0.13

A discriminant analysis was performed to distinguish between the guilty and informed groups in the P3-based GKT, RM, and combination of the two. The discriminant function analysis results for the P3-based GKT were significant, χ^2^_(3, *N* = 30)_ = 25.53, *p* < 0.001, and showed an 86.7% overall correct classification accuracy. A total of 86.7% or the guilty group and 86.7% of the informed group were classified correctly. An internal validation of the discriminant analysis also indicated 86.7% correct classifications. For the results of the RM, the discriminant function indicated significant differences, χ^2^_(5, *N* = 30)_ = 23.57, *p* < 0.001. The mean classification accuracy was 86.7%. For these analyses, 86.7% of the guilty group and 86.7% of the informed group were classified correctly. An internal validation of the discriminant analysis showed 76.7% correct classifications, and 66.7% of the guilty group and 86.7% of the informed group were correctly classified. The result of a combination between the P3-based GKT and RM showed significant differences in the predicted variables, χ^2^_(8, *N* = 30)_ = 34.22, *p* < 0.001. The overall classification accuracy was 93.3%. In this result, 86.7% of the guilty group and 100.0% of the witness group were classified correctly both in the present study. An internal validation of the discriminant analysis was 90.0% (Table 3).

**Table 3:** **Discriminant analyses with P3-based GKT and reality monitoring between the guilty and informed group**.

**Cross validated classification rates (original classification)**					
	**Hit rates**			**Eigenvalue**	**λ**
	**Guilty group**	**Informed group**	**Total**		
P3-GKT	86.7 (86.7)	86.7 (86.7)	86.7 (86.7)	1.62	0.38
RM	66.7 (86.7)	86.7 (86.7)	76.7 (86.7)	1.52	0.40
P3-GKT + RM	86.7 (86.7)	93.3 (100.0)	90.0 (93.3)	3.16	0.24

## Discussion

The purpose of this study was to investigate differences in the P3-based GKT or RM among individuals in the guilty, witness, and informed groups. Additionally, we investigated whether the combination of the P3-based GKT and RM would more accurately discriminate among the groups than either test alone.

The results indicated that the informed group showed lower P3 amplitude in response to the probe stimulus than did the guilty and witness groups. These results partly support the first hypothesis. Indeed, the results suggest that the P3-based GKT may differentiate individuals who do not experience the crime but who have information about the crime (i.e., informed individuals), from those who do experience it (i.e., witnesses, and guilty individuals). The informed individuals may have less specific memories surrounding they crime compared to individuals who experience the crime because they did not directly experience the crime. It may be difficult to identify the source of their crime-related knowledge (Zvi et al., 2012). More specifically, the guilty and witness groups recognized the assailants' better than the informed group, presumably because these two groups had direct experiences with the accident. The P3-based GKT, however, did not reveal significant differences between the guilty and witness groups regarding responses to the probe stimuli. Given these findings, the P3-based GKT appears to be weak in terms of its ability to discriminate between groups when knowledge of a crime is leaked (Ben-Shakhar and Elaad, 2003). The witness and guilty groups may have had similar amounts of vivid information about the crime stored in memory. If this is the case, the P3-based GKT may not have be able to differentiate between the guilty and witness groups. Thus, the P3-based GKT may need another tool to overcome this weakness. More specifically, another tool is needed to differentiate between guilty individuals and witnesses.

Regarding the RM, there were significant differences among the groups, in the visual, temporal, and spatial details as well as cognitive operations. The statements from the witness group included more visual details, temporal information, spatial information, and less cognitive operations than those from the guilty and informed groups. These results may be due to differences in the quality of the crime-related information stored in memory (Vrij et al., 2004). Most likely, the witness group recalled the sensory, perceptual, and contextual memories that they experienced in the VE. The guilty and informed groups, however, described a story that they did not experience. More specifically, the guilty group had to provide a false statement. The informed group had to provide factual statements about the accident. Thus, they were less likely to have limited sensory, perceptual, and contextual memories of the accident. These results suggest that RM may differentiate the experienced-driven true memories and false or imagined memories. Given these findings, the RM seems to be weak in terms of its ability to accurately distinguish among the three groups.

The discriminant analyses revealed that the combination of the P3-based GKT and RM showed higher accuracy rates compared to two methods independently. In terms of the results of the P3-based GKT, the discriminate function was unable to distinguish between the guilty individuals and witnesses, whereas it was able to distinguish between the guilty and informed individuals. These results indicate that the P3-based GKT may not differentiate between witnesses and guilty individuals. Thus, the results suggest the P3-based GKT is weak when knowledge of a crime is leaked. In other words, the witnesses may be falsely accused of committing a crime when the P3-based GKT is employed for the purpose of lie detecting. The results pertaining to RM analysis revealed a high discrimination rate for the witness group (100.0% of the witness group). The combination of the P3-based GKT and RM correctly classified 100.0% of the witness group. This result implies that RM may overcome a weakness of the P3-based GKT. Additionally, the discriminant analysis of the P3-based GKT revealed that it was highly able to discriminate between the guilty and informed individuals, whereas the discriminant analysis of the RM showed moderate discrimination (an internal validation of discrimination analysis: 76.7%). These results highlight a limitation of RM because both the guilty and informed individuals should possess an imagined memory of the crime that is reflected in their statement. Regarding the overall classification rate, the combination of the P3-based GKT and RM also showed a higher rate of classifications than either the P3-based GKT or RM alone, although differences in correct-classification rates among the techniques were not examined. The results of the present study are comparable to a previous study in which the combination of the Criteria-Based Content Analysis and RM correctly classified 80.8% of the participants (Vrij et al., 2000). However, the aim of the previous study was to discriminate between the guilty group and innocent group that had no information about the crime. In the present study, the combined method showed a higher classification rate (an internal validation between the guilty and witness group of 100.0% and an internal validation between the guilty and informed group of 90.0%). By combing the P3-based GKT and RM, each method builds upon the weaknesses of the other method. In conclusion, the present study suggests that the combination of the P3-based GKT and RM may differentiate among individuals who are guilty, witnesses, and informed.

In the present study, the RM was used to build upon a weakness of the P3-based GKT. Although the combination of the P3-based GKT and RM was adequately able to differentiate between the guilty, witness, and informed groups, this combination also has some possible weakness. If the guilty individuals know the RM criteria, they may be able to manipulate the quality of their report by intentionally changing the balance among cognitive operations, visual, auditory, temporal, and spatial details. Therefore, future studies need to identify the optimal combination of the P3-based GKT and other various methods to differentiate between the guilty individuals and the witnesses or informed individuals.

The present study has several implications. First, the present study suggests that a combination of the P3-based GKT and RM may build upon the weakness of the P3-based GKT because the test is susceptible to the leakage of information about the crime. Therefore, the method may help protect innocent individuals from perceived as guilty when they have information about the crime that was disclosed to the public through mass media or by participating in the investigation.

Second, the present study showed that the GKT using the image stimulus that participants experienced in a VE can discriminate between the groups. Previous study of ERP-based deception detection using a mock crime in a VE indicated that the hit rates were quite low (Mertens and Allen, 2008). Possible reason for such low hit rates would be due to the feature of stimulus. For example, they used stimulus consisting of words, but not images. Many studies have suggested the picture superiority effect (Buckner et al., 2000) that pictures are better recalled than words and better recollected when cued with a fragment only (McBride and Dosher, 2002; Cutmore et al., 2009). Given these, it appears to be reasonable to use image stimuli for a GKT in a VE than word stimuli. Although the findings on the detection of deception using the ERPs-based GKT in a VE have been acceptable in a laboratory experiment, we suggest that future research apply the ERPs-based GKT in a real forensic situation. For example, in a real forensic situation, there could be delay between conducting a crime and assessing deception. However, in the laboratory setting, the deception is assessed right after conducting a mock crime. Therefore, we suggest that future studies compare memories about a crime both immediately after the crime and after a delayed period.

### Conflict of interest statement

The authors declare that the research was conducted in the absence of any commercial or financial relationships that could be construed as a potential conflict of interest.
